# In Vitro evaluation of the effects of whitening toothpastes on the color and surface roughness of different composite resin materials

**DOI:** 10.1186/s12903-023-03277-4

**Published:** 2023-08-19

**Authors:** Gulben Colak, Gunseli Katirci

**Affiliations:** Faculty of Dentistry, Department of Restorative Dentistry, Suleyman Demirel University, Isparta, Turkey

**Keywords:** Whitening toothpaste, Composite resin, Discoloration, Tooth brushing, Surface roughness

## Abstract

**Background:**

The aim of this study was to evaluate the effects of traditional and whitening toothpastes on the color and surface roughness of different composite resin materials.

**Methods:**

Eighty disc-shaped samples were prepared for each of the following composite resins: nano-hybrid (Filtek Ultimate Universal; 3 M/ESPE, Saint Paul, USA), micro-hybrid (Charisma Smart; Kulzer, Hanau, Germany) and supra-nano-filled (Omnichroma; Tokuyama, Tokyo, Japan). Each composite-resin sample was randomly divided into the following four subgroups (*n* = 20 per group): *Group 1*, control; *Group 2*, traditional toothpaste (Colgate Total 12; Colgate Palmolive, New York, USA); *Group 3*, peroxide-based toothpaste (Colgate Optic White; Colgate-Palmolive, New York, USA); and *Group 4*, blue covarine-based toothpaste (Meridol Gentle White; CP-GABA, Hamburg, Germany). The samples for the toothpaste subgroups were immersed in a coffee solution for 10 min and washed twice a day before each brushing cycle. The specimens were brushed for 30 days. Color analyses were performed using a spectrophotometer (SpectroShade Micro, MHT, Italy). Surface roughness analyses were conducted using a profilometer (Surftest SJ-210 Mitutoyo, Tokyo, Japon). The color and surface roughness analyses were performed at baseline and 1, 7 and 30 days after each treatment. Furthermore, surface topography analysis was performed using Scanning Electron Microscopy (FEG 250-FeiQuanta, the Netherlands). The data were analysed with a three-way robust ANOVA and Bonferroni post-hoc correction (*p* < 0.05).

**Results:**

The smallest color change was observed for the micro-hybrid composite resin, and the greatest color change was observed for the nano-hybrid composite resin. Based on the tested composite resin samples, the greatest color change was obtained after using blue covarine–based toothpaste, while the smallest color change was observed after using peroxide-based toothpaste. Moreover, the supra-nano-filled composite resin samples exhibited the lowest roughness values (robust ANOVA test, *p* < 0.001). There was no statistically significant difference between the mean values of roughness for the composite, group and time interaction (*p* = 0.937).

**Conclusion:**

Charisma Smart composite resin exhibited significantly lower staining than all the other composite resins tested after using all toothpastes included in the study. Further laboratory and clinical studies are needed to fully understand the long-term effectiveness of whitening toothpaste on composite resin materials.

## Introduction

The desire for aesthetic improvement has led to the introduction of numerous materials and methods for teeth whitening and restoration in dentistry [1, 2]. Some people choose home-whitening techniques, including the use of whitening toothpaste, due to favourable bleaching results. Whitening toothpaste provides satisfactory results over a short period of time [3]. This kind of toothpaste usually contains whitening agents and abrasives that can remove extrinsic stains from teeth in a fast, suitable and cheap manner [3–5]. Hydrogen peroxide and carbamide peroxide are frequently used as whitening agents in different concentrations. Teeth whitening occurs due to oxy-reduction reactions, as pigments are reduced into smaller molecules with peroxide-containing toothpaste [3, 6]. Furthermore, extrinsic stains can be removed with abrasives. During brushing, abrasive particles become lodged between the bristles of the toothbrush and the surfaces of the teeth. The stains are removed due to the hardness of the abrasive, thus cleaning the surface of the tooth. However, only the extrinsic stains of the tooth are affected by this mechanism rather than the natural tooth color or internal discoloration [3, 7–9]. At the same time, optical modifying toothpastes contain pigments, such as blue covarine, which can change the apparent color of teeth by depositing a thin, semi-transparent film of bluish pigment on the dental surface. This film modifies the interaction of incident light, making teeth appear whiter and brighter [7, 10–13].

The color change of composite resin may be affected by factors such as matrix type, filler type and coloring agents. Discoloration of composite resin can intensify due to contact with staining agents or alcoholic and acidic media, which are present in the diets of most people, further degrading the organic matrix. Roughness is another factor that may cause composite resin discoloration. The surface characteristics of composite resins can be improved using high-quality finishing and polishing methods, as rough surfaces may cause discolorations, plaque accumulation, recurrent caries and gingival irritations, in addition to producing inconvenience and cleaning difficulties [14].

The literature has reported that whitening toothpastes with abrasive particles may produce roughness on the surfaces of composite resin materials [15]. It has been noted that increasing porosity on the surface can change the composite resin by causing volumetric loss or high water withdrawal [16]. During brushing, the polymer matrix of the composite resin can degrade and change the surface hardness of the composite resin, subsequently enhancing pigmentation [8, 17, 18]. Moreover, the composite resin surface remains rough because the bristles of the toothbrush cannot abrade and smoothen fillers in the way that the rubber core or disc used for polishing the material can [8]. Abrasion can lead to changes in the surface of materials, thus affecting the contours and colors of teeth due to surface roughness [18].

In the literature, several clinical and laboratory studies have evaluated the effects of whitening toothpastes on the discoloration and surface roughness of composite resin [9, 16]. However, the recent development of new agents and materials means that our knowledge of this topic is limited [16]. Therefore, the aim of this in vitro study was to evaluate the effects of traditional, peroxide-based and blue covarine-based toothpastes on the color change and surface roughness of nano-hybrid, micro-hybrid and supra-nano-filled composite resin materials. In addition, we analysed the morphological changes in the composite resin surfaces by using scanning electron microscopy (SEM) after the application of the toothpastes. The null hypothesis was that there would be no differences in color change and surface roughness of the nano-hybrid, micro-hybrid and supra-nano-filled composite resin samples after brushing with the selected whitening toothpastes.

## Materials and methods

We used three direct restorative materials. Details of the composite resin materials used in the study are provided in Table 1.

**Table 1:** The restorative materials used in the study are according to composition and shade selection

Product name	Composite resin type	Composition	Filler content		Shade	LOT number	Manufacturer
			**wt. (%)**	**vol. (%)**			
Filtek Ultimate Universal	Nano-hybrid	Bis-GMA, Bis-EMA, UDMA, TEGDMA, PEGDMA resins and zirconia/silica	78.5	63.3	A2E	NA41297	3 M ESPE, Saint Paul, USA
Charisma Smart	Micro-hybrid	Bis-EMA, HEDMA, TEGDMA, Barium Aluminium Fluoride glass (0.02–2 μm), pyrogenic silicon dioxide (0.02–0.07 μm)	78	65	A2	K010527	Kulzer, Hanau, Germany
Omnichroma	Supra-nano-filled	UDMA, TEGDMA, uniform-sized supra-nano spherical filler (260 nm spherical SiO2-ZrO2), composite filler (SiO2-ZrO2)	79	68	Single shade	045EZ0	Tokuyama, Tokyo, Japan

### Sample size calculation

The software G*Power (G*Power Ver. 3.0.10, Germany) was used to determine the minimum sample size, with 95% statistical significance and 0.80 test power in 1.0 effect size. For this study, the calculated minimum sample size was 17. To prevent possible data loss, three samples were added to each group, as we decided that the study would be performed with 20 samples for each group.

### Sample preparation

Eighty disc-shaped samples were created for each composite resin using a Teflon matrix (2 mm in height and 10 mm in diameter). Filtek Ultimate Universal and Charisma Smart composite resin materials were used in the A2 shade for standardisation. Omnichroma composite resin was a single-shade material. The specimens were cured with a halogen curing unit at a light intensity of 400–550 mW/cm^2^ and using a standard curing mode (Demetron LC, Kerr, Collins Ave., USA) for each side for 20 s according to the instructions of the manufacturer. The intensity of the light-curing unit was verified with a calibrated radiometer for every five specimens.

A mylar strip was placed over the composite resin and pressed using a glass plate removed after curing to provide a flat surface. The samples were polished using an electric handpiece at 15,000 rpm and a series of polishing discs (Super-Snap, Rainbow Technique Kit, Shofu Inc, Kyoto, Japan) for 10 s (coarse, medium, fine and superfine). The thickness of each sample was measured with a digital caliper (Mitutoyo, Kawasaki, Japan) for standardisation. After polishing, the specimens were kept in deionized water at 37 °C for 24 h [19].

Then, the samples were kept in artificial saliva (0.33 g of KH2PO4, 0.34 g of Na2HPO4, 1.27 g of KCl, 0.16 g of NaSCN, 0.58 g of NaCl, 0.17 g of CaCl2, 0.16 g of NH4Cl, 0.03 g of glucose, 0.2 g of urea, 0.002 g of ascorbic acid and 2.7 g of mucin in 1000 mL of distilled water) in an incubator (FN055-Nuve, Ankara, Turkey) at 37 °C for 24 h.

The sample for each composite resin (*n* = 80) was randomly divided into the following four subgroups (*n* = 20 per group) based on the type of toothpaste used in the study:

Group 1. For the control group, the samples were stored in artificial saliva at 37 °C throughout the study period. The artificial saliva was changed every day.

Group 2. The samples were brushed with Colgate Total 12 (Colgate Total 12, Colgate-Palmolive, New York, USA) toothpaste after immersion in a coffee solution twice a day for 10 min for 30 days.

Group 3. The samples were brushed with Colgate Optik White toothpaste (Colgate Optic White, Colgate-Palmolive, New York, USA) after immersion in a coffee solution twice a day for 10 min for 30 days.

Group 4. The samples were brushed with Meridol Gentle White toothpaste (Meridol Gentle White, CP-GABA, Hamburg, Germany) after immersion in a coffee solution twice a day for 10 min for 30 days.

During resting periods, the samples were stored in artificial saliva at 37 °C, and the artificial saliva was changed every day during the study period.

### Calibration

All of the color and surface roughness analyses of the samples were performed by one investigator (GC). She was trained by using a spectrophotometer and profilometer [20]. To validate, the investigator measured the color and surface roughness of the ten samples from each composite resin material that was not included in the study. The analysis was performed two times with an interval of one week between two measurements. This resulted in an intra-agreement rate of 98%, considered high reproducibility.

### Blinding

According to a list constituted by RANDOM.ORG, all specimens of each composite resin group were numbered and randomly assigned into four subgroups. The sequence of toothpaste application was randomized with the same technique [20].

### Staining in coffee solution and brushing simulation

For each daily brushing cycle, the samples were first immersed in 2 ml of coffee solution at 37 ºC for 10 min and then washed with distilled water. The coffee solution was constructed using 1 tsp of soluble coffee (Nescafe Original, Nestle, Araras, Sao Paulo, Brazil) dissolved in 50 ml of boiling water [18].

The specimens in the brushing subgroups were also brushed with toothpaste twice a day using an electric toothbrush (Oral B Vitality Plus 2D Cross Action, Germany) for five seconds on each surface for 30 days. We chose five seconds per sample because that is the typical amount of time that a person brushes a stained surface [21, 22]. The horizontal brushing technique was used by one investigator (GC) to eliminate potential differences among investigators and standardize the brushing force. The toothpaste was diluted in distilled water every brushing cycle (1:3) (in weight) [20].

After filtering the liquids of the specimens, gently rinsing them with distilled water, and drying them with paper tissue, we performed color measurements. The toothpastes used in this study are presented in Table 2.

**Table 2:** Toothpastes were used in the study according to composition

Toothpaste	RDA	Composition	Major whitening mechanism	Manufacturer
Colgate Total 12	70	Water, sodium fluoride, triclosan, hydrated silica, glycerine, sorbitol, PVM/MA copolymer, sodium lauryl sulphate, flavour, cellulose, sodium hydroxide, propylene glycol, carrageenan, sodium saccharin, titanium dioxide	-	Colgate-Palmolive, New York, USA
Colgate Optik White	100	Water, propylene glycol, calcium phosphate, PVP, PEG/PPG-116/66 copolymer, PEG-12, glycerine, flavour, hydrogen peroxide, sodium lauryl sulphate, silica, tetrasodium pyrophosphate, sodium saccharin, disodium pyrophosphate, sucralose, phosphoric acid, BHT, hydrogen peroxide	Hydrogen peroxide	Colgate-Palmolive, New York, USA
Meridol Gentle White	75	Aqua, hydrated silica, glycerine, hydroxyethyl cellulose, aroma, cocamidopropyl betaine, sodium gluconate, PEG-3, tallow aminopropyl amine, olafluor, stannous fluoride, sodium saccharin, hydrochloric acid, potassium hydroxide, limonene, CI 77,891, CI 7416 (blue covarine)	Blue covarine	CP-GABA, Germany

**Table 3a:** Descriptive statistics and multiple comparison results for L* values according to composite resin, group and time

Group	Time	Composite resin materials			Total
		FILTEK	CHARISMA	OMNICHROMA	
**Group 1**	Baseline 1 day 7 days 30 days Total	68.3 ± 0.1^ A^ 68.2 ± 0.2^AB^ 66.3 ± 0.3^CDEFGHİJKLM^ 67.7 ± 0.3^ABCDEFJ^ 67.7 ± 0.1^AB^	68.0 ± 0.4^ABCDEFGJM^ 68.1 ± 0.3^ABCF^ 68.0 ± 0.3^ABCF^ 68.3 ± 0.2^ A^ 68.1 ± 0.1^ A^	65.0 ± 0.1^KOWZ.(AA)^ 64.1 ± 0.2^QSWZ.(AA)^ 63.9 ± 0.2^QSWZ^ 64.9 ± 0.1^KOWZ.(AA)^ 64.5 ± 0.1^ C^	67.3 ± 0.3^ABC^ 67.0 ± 0.4^ABC^ 66.1 ± 0.3^AB^ 67.1 ± 0.3^ABC^ 66.9 ± 0.2^a^
**Group 2**	Baseline 1 day 7 days 30 days Total	68.4 ± 0.1^ A^ 65.9 ± 0.2^GHİKLNO^ 61.2 ± 0.6^PQRSTUVW^ 54.3 ± 1.3^PTUVXY^ 63.6 ± 0.7^CD^	67.8 ± 0.2^ABC^ 67.5 ± 0.1^ABCD^ 66.5 ± 0.2^CDEFGHJM^ 63.3 ± 0.3^QRSWZ^ 66.7 ± 0.2^BF^	65.5 ± 0.4^DEGHİJKLMNOZ.(AA)^ 64.6 ± 0.3^KLNOQWZ.(AA)^ 62.5 ± 0.2^PR^ 58.6 ± 0.2^TUX^ 63.1 ± 0.4^CD^	67.6 ± 0.2^ C^ 66.1 ± 0.2^ A^ 63.4 ± 0.4^D^ 59.1 ± 0.6^E^ 64.7 ± 0.3^b^
**Group 3**	Baseline 1 day 7 days 30 days Total	68.1 ± 0.2^AB^ 66.5 ± 0.2^CDEFGHİJM^ 61.6 ± 0.5^PQRSTV^ 55.0 ± 0.9^XY^ 64.0 ± 0.8^CDEF^	67.6 ± 0.1^ABCD^ 66.9 ± 0.2^BCDEFGJM^ 66.0 ± 0.1^EGHİLN^ 64.3 ± 0.3^İKLNOQRSWZ.(AA)^ 66.4 ± 0.2^EF^	65.3 ± 0.2^HİKLNO.(AA)^ 64.7 ± 0.2^KLNOWZ.(AA)^ 62.5 ± 0.2PRS 58.2 ± 0.2UX 63.2 ± 0.5CD	67.4 ± 0.2^BC^ 66.2 ± 0.2^ A^ 63.4 ± 0.4^D^ 59.2 ± 0.7^E^ 64.8 ± 0.3^b^
**Group 4**	Baseline 1 day 7 days 30 days Total	68.6 ± 0.2^ A^ 65.8 ± 0.2^GHİKLMNO^ 61.9 ± 0.6^NOPQRSTUVWZ.(AA)^ 54.4 ± 1.0^X^ 63.7 ± 0.8^CDE^	68.0 ± 0.1^ A^ 67.1 ± 0.1^BCDFJM^ 66.3 ± 0.1^EFGHİJM^ 64.5 ± 0.4^HİKLNOQRSWZ.(AA)^ 66.7 ± 0.2^ F^	65.0 ± 0.2^HİKLNOWZ.(AA)^ 64.0 ± 0.3^NOQRSWZ.(AA)^ 60.9 ± 0.2^VY^ 56.8 ± 0.3^X^ 62.2 ± 0.5^D^	67.6 ± 0.3^ C^ 66.0 ± 0.3^ A^ 62.7 ± 0.5^D^ 58.3 ± 0.8^E^ 64.5 ± 0.3^b^
**Total**	Baseline 1 day 7 days 30 days Total	68.3 ± 0.1^ A^ 66.4 ± 0.1^B^ 62.6 ± 0.4^ C^ 57.2 ± 1.1^D^ 65.3 ± 0.3^a^	67.8 ± 0.1^E^ 67.4 ± 0.1^E^ 66.5 ± 0.1^B^ 64.7 ± 0.3^FG^ 67.0 ± 0.1^b^	65.1 ± 0.1^ F^ 64.3 ± 0.1^G^ 62.5 ± 0.2^ C^ 58.9 ± 0.5^D^ 63.6 ± 0.2^c^	67.5 ± 0.1^a^ 66.2 ± 0.1^b^ 64.0 ± 0.2^c^ 61.1 ± 0.4^d^ 65.4 ± 0.1

a–d: No difference between the main effects with the same letter; A–(AA): No difference between interactions that have the same letter; pruned average ± standard error

### Color measurement

The color analysis of the samples was performed by a blind-trained investigator at baseline (before the staining) and 1 day, 7 days and 30 days after the brushing to determine the color change rate. To avoid the influence of light in the environment, the measurements were performed in a windowless dark laboratory.

The color analyses of the composite resin samples were conducted using a spectrophotometer (SpectroShade Micro, MHT, Italy) according to the CIE L*a*b* coordinates that help identify the color of an object in a three-dimensional color space. The chromaticity coordinates are the axes of a* and b*. The L* axis, perpendicular to a* and b*, shows the perceived color lightness.

Before all measurements, the spectrophotometer was calibrated according to manufacturer recommendations. The measuring tip of the instrument was placed at a right angle to the sample surface and at the same distance each time. The color of each sample was measured three times. The images of the samples were then opened and saved using the device’s computer software (Spectroshade Micro-Software-Version 3.01, MHT, Italy). Measurements were taken based on the images, and the samples’ L*, a*, and b* values were recorded. The L* parameter (white-black range) demonstrated the brightness of the samples, the a* parameter (red-green range) demonstrated the redness and the b* parameter demonstrated the yellowness (yellow-blue range).

The color changes (ΔE_00_) of the samples were calculated using the CIEDE2000 formula: ΔE_00_ = [(ΔL´/K_L_S_L_)^2^ + (ΔC´/K_C_S_C_)^2^ + (ΔH´/K_H_S_H_)^2^ + RT(ΔC´/K_C_S_C_) X (ΔH´/K_H_S_H_)^2^]^1/2^, where ΔL´, ΔC´, and ΔH´ are the distinctions in terms of lightness, chroma and hue, respectively, between the two samples in CIEDE2000. RT is the rotation function that accounts for the interaction between chroma and hue differences in the blue zone. S_L_, S_C_ and S_H_ are the weighting functions that adjust the total color difference for variation according to the location of the color difference pair at L*, a*, and b* coordinates. The parametric factors (K_L_, K_C_ and K_H_) are correction terms for experimental conditions, as described by Sharma et al. [1, 23].

The color changes were calculated between baseline and 1 day [ΔE_00 (1 day−baseline)_], 7 days [ΔE_00 (7 days−baseline)_] and 30 days [ΔE_00 (30 days−baseline)_] after brushing.

### Surface roughness measurement

Surface roughness analyses of the composite resin samples were conducted using a profilometer (Surftest SJ-210 Mitutoyo, Tokyo, Japon). The values were recorded at the baseline and 1 day, 7 days and 30 days after brushing with the toothpaste to evaluate the rate of surface roughness. The measurements were performed at a speed of 0.25 mm/sn and with a cut-off of 0.80 mm. Three readings were taken for each sample, and the averages of these values (Ra, µm) were recorded across the four evaluation periods.

### Surface topography measurement

After 30 days of brushing with different kinds of toothpaste, two samples were randomly selected from each composite resin subgroup to analyse their surface topography. The analysis was performed using SEM (FEG 250-FeiQuanta, the Netherlands). The most representative images were archived for illustration.

### Statistical analysis

The data were analysed using the R programme. The normality distribution of the data was analysed using the Shapiro-Wilks test. The WRS2 package was used to compare non-normal distributed values of color and roughness according to the composite resin, group and time. The data were examined using a three-way robust analysis of ANOVA with a pruned mean. Multiple comparisons were performed using Bonferroni post hoc correction. The significance level was *p* < 0.05 for all tests.

## Results

### Color analysis

The mean values and standard deviations for L*, a*, b* and ΔE_00_ are presented in Table 3a, 3b, 3c and 3d. We found a statistically significant difference between the L*, a* and b* values according to composite resin, group and time (*p* < 0 0.01, *p* < 0.01 and *p* = 0.001) (Table 4).

Charisma had the highest L* (67.0) and a* (1.49) values, while Omnichroma had the lowest L* (63.6) and a* (-4.00) values (Tables 3a and 3b). The b* value for Filtek (17.2) was higher than the b* values for Charisma (16.3) and Omnichroma (8.2) (Table 3c). Whereas the smallest color change (ΔE_00_) was observed for Charisma (1.81), the greatest color change was exhibited by Filtek (5.18) (Table 3d). Moreover, we found that the highest L* values and the lowest a* and b* values belonged to the control group for all the tested composite resin materials. The L* values decreased and the a* and b* values increased over time for all the tested composite resin materials.

The greatest color change (ΔE_00_) occurred in Group 4 (4.73) and after four weeks (6.47) for all the tested composite resin materials (Table 3d). Furthermore, we observed a statistically significant difference between L*, a*, b* and ΔE_00_ values in terms of the composite resin, group and time interaction (*p* = 0.01) (Table 4). The highest mean L* value was obtained by Group 4 of Filtek at baseline (68.6), while the lowest mean L* value was obtained by Group 2 of Filtek (54.3) after four weeks (Table 3a). The greatest color change (ΔE_00_) was observed for Group 2 of the Filtek after 30 days (Table 3d).

### Surface roughness analysis

The mean values and standard deviations of the results for the surface roughness values are presented in Table 5. We found statistically significant differences between the surface roughness values according to composite resin, group and time (Table 6). Omnichroma (0.167) exhibited lower roughness values than Charisma (0.230) and Filtek (0.235). The highest surface roughness values were obtained by Group 4 (0.225) and after four weeks (0.226) by all test groups (Table 5). Furthermore, there was no statistically significant difference between the mean values of roughness according to the composite resin, group and time interaction (*p* = 0.937) (Table 6).

**Table 3b:** Descriptive statistics and multiple comparison results for a* values according to composite resin, group and time

Group	Time	Composite resin materials			Total
		FILTEK	CHARISMA	OMNICHROMA	
**Group 1**	Baseline 1 day 7 days 30 days Total	-1.13 ± 0.02^AB^ -1.09 ± 0.11^AB^ -0.98 ± 0.01^ A^ -0.98 ± 0.01^ A^ -1.03 ± 0.02^ A^	1.17 ± 0.21^DFGHİJK^ 0.98 ± 0.00^ F^ 0.98 ± 0.00^ F^ 0.98 ± 0.00^ F^ 0.98 ± 0.00^B^	-4.45 ± 0.05^ L^ -4.40 ± 0.11^ L^ -4.46 ± 0.04^ L^ -4.45 ± 0.03^ L^ -4.44 ± 0.03^D^	-1.46 ± 0.47^ A^ -1.25 ± 0.47^ A^ -1.27 ± 0.48^ A^ -1.28 ± 0.48^ A^ -1.32 ± 0.24^a^
**Group 2**	Baseline 1 day 7 days 30 days Total	-1.15 ± 0.03^AB^ -0.15 ± 0.06^CDE^ 1.38 ± 0.27^CDFGHİJK^ 3.47 ± 0.63^CDEFGHİJK^ 0.56 ± 0.26^B^	1.23 ± 0.05^FG^ 1.59 ± 0.06^GHİJ^ 1.82 ± 0.07^HİK^ 2.31 ± 0.09^ K^ 1.69 ± 0.06^ C^	-4.59 ± 0.07^ L^ -4.40 ± 0.07^ L^ -3.58 ± 0.08^ M^ -2.19 ± 0.09^ N^ -3.82 ± 0.15^E^	-1.43 ± 0.48^ A^ -0.71 ± 0.52^AB^ 0.30 ± 0.50^AB^ 1.35 ± 0.46^B^ -0.10 ± 0.23^b^
**Group 3**	Baseline 1 day 7 days 30 days Total	-0.64 ± 0.09^AE^ 0.28 ± 0.07^CD^ 1.73 ± 0.15^FGHİJK^ 3.24 ± 0.39^FGHİJK^ 1.02 ± 0.22^BC^	1.34 ± 0.05^GJ^ 1.54 ± 0.03^GHJ^ 1.98 ± 0.05^İK^ 2.23 ± 0.08^ K^ 1.72 ± 0.06^ C^	-4.62 ± 0.06^ L^ -4.36 ± 0.05^ L^ -3.61 ± 0.08^ M^ -1.82 ± 0.09^ N^ -3.69 ± 0.18^E^	-0.92 ± 0.50^AB^ -0.40 ± 0.53^AB^ 0.53 ± 0.53^AB^ 1.47 ± 0.42^B^ 0.21 ± 0.23^b^
**Group 4**	Baseline 1 day 7 days 30 days Total	-1.24 ± 0.04^B^ -0.20 ± 0.09^CE^ 1.43 ± 0.26^DFGHİJK^ 3.05 ± 0.36^FGHİJK^ 0.57 ± 0.26^B^	1.14 ± 0.06^FG^ 1.66 ± 0.05^HİJ^ 1.85 ± 0.03^İK^ 2.04 ± 0.05^ K^ 1.69 ± 0.05^ C^	-4.48 ± 0.04^ L^ -4.23 ± 0.04^ L^ -3.04 ± 0.09^ M^ -1.58 ± 0.08^BN^ -3.47 ± 0.19^E^	-1.47 ± 0.47^ A^ -0.67 ± 0.51^AB^ 0.53 ± 0.45^AB^ 1.33 ± 0.38^B^ 0.04 ± 0.21^b^
**Total**	Baseline 1 day 7 days 30 days Total	-1.10 ± 0.03^ A^ -0.19 ± 0.06^B^ 1.03 ± 0.21^CD^ 2.31 ± 0.38^CDE^ 0.07 ± 0.11^a^	1.23 ± 0.04^ C^ 1.41 ± 0.06^CD^ 1.69 ± 0.08^DE^ 1.95 ± 0.10^E^ 1.49 ± 0.04^b^	-4.52 ± 0.03^ F^ -4.33 ± 0.03^G^ -3.61 ± 0.07^ H^ -2.22 ± 0.20^İ^ -4.00 ± 0.07^c^	-1.32 ± 0.24^a^ -0.80 ± 0.25^ab^ 0.00 ± 0.24^bc^ 0.71 ± 0.20^c^ -0.32 ± 0.12

a–c: No difference between the main effects with the same letter; A–N: No difference between interactions that have the same letter; pruned average ± standard error

**Table 3c:** Descriptive statistics and multiple comparison results for b* values according to composite resin, group and time

Group	Time	Composite resin materials			Total
		FILTEK	CHARISMA	OMNICHROMA	
**Group 1**	Baseline 1 day 7 days 30 days Total	15.0 ± 0.2^ABCDEFGHİJKL^ 15.1 ± 0.0^ABDEFGİJKM^ 15.0 ± 0.0^ABDEFGİJKM^ 15.1 ± 0.1^ABDEFGİJKM^ 15.1 ± 0.0^ A^	16.0 ± 0.2^ABCGHLMO^ 16.0 ± 0.2^ABCGHLMO^ 15.8 ± 0.1^CHLO^ 15.9 ± 0.0^CHLO^ 15.9 ± 0.1^D^	6.0 ± 0.3^ST^ 6.0 ± 0.0^ S^ 5.8 ± 0.1^ S^ 6.0 ± 0.1^ S^ 5.9 ± 0.0^E^	13.0 ± 0.9^ABCD^ 13.0 ± 1.0^ABCD^ 13.2 ± 0.9^ABCDE^ 13.3 ± 0.9^ABCDE^ 13.1 ± 0.5^a^
**Group 2**	Baseline 1 day 7 days 30 days Total	15.0 ± 0.2^ACDEFGHİJK^ 17.6 ± 0.4^BLMNOP^ 22.0 ± 1.1^ABCLMNOPQ^ 25.8 ± 1.8^ABCDELMNOPQ^ 19.0 ± 0.7^B^	15.6 ± 0.3^ABCDEFGHİJKLMO^ 16.3 ± 0.3^ABCDEFGHLMO^ 16.9 ± 0.4^ABCDELMNO^ 17.1 ± 0.5^ABCDELMNOP^ 16.4 ± 0.2^CD^	6.3 ± 0.2^ST^ 8.7 ± 0.2^U^ 12.0 ± 0.4^RV^ 13.4 ± 0.5^FGHİJKRV^ 10.0 ± 0.4^ F^	12.9 ± 0.8^AC^ 14.8 ± 0.8^ABCDE^ 16.6 ± 0.7^ABCDE^ 17.5 ± 0.9^BDE^ 15.4 ± 0.3^b^
**Group 3**	Baseline 1 day 7 days 30 days Total	14.7 ± 0.1^DEFİJKR^ 16.5 ± 0.5^ABCDEFGHİJKLMNO^ 21.1 ± 0.7^NPQ^ 24.6 ± 1.0^Q^ 18.5 ± 0.7^BC^	15.6 ± 0.2^ABCDEFGHJLMO^ 16.0 ± 0.2^ABCGHLMO^ 17.1 ± 0.4^ABCELMNOP^ 17.7 ± 0.6^ABCDELMNOP^ 16.4 ± 0.2^CD^	6.1 ± 0.3S^T^ 7.7 ± 0.2^TU^ 11.4 ± 0.4^ V^ 12.9 ± 0.4^JKRV^ 9.4 ± 0.4^ F^	13.0 ± 0.9^ABC^ 14.0 ± 0.8^ABCDE^ 16.5 ± 0.7^ABCDE^ 17.9 ± 0.9^E^ 15.3 ± 0.3^b^
**Group 4**	Baseline 1 day 7 days 30 days Total	14.6 ± 0.1^DFİJKR^ 17.2 ± 0.6^ABCDEFGHLMNOP^ 21.5 ± 1.1^ABCDELMNOPQ^ 24.1 ± 1.1^PQ^ 18.6 ± 0.7^BC^	15.2 ± 0.3^ABCDEFGHİJKLMO^ 16.0 ± 0.2^ABCDEFGHLMO^ 17.3 ± 0.3^MNOP^ 17.4 ± 0.7^ABCDEFGHİLMNOP^ 16.4 ± 0.2^CD^	5.7 ± 0.1^ S^ 7.7 ± 0.3^TU^ 11.8 ± 0.3^ V^ 13.3 ± 0.3^İKRV^ 9.4 ± 0.5^ F^	12.5 ± 0.9^ A^ 14.2 ± 0.8^ABCDE^ 16.7 ± 0.7^BCDE^ 17.5 ± 0.8^DE^ 15.3 ± 0.3^b^
**Total**	Baseline 1 day 7 days 30 days Total	14.8 ± 0.1^ A^ 16.4 ± 0.3^BC^ 19.7 ± 0.7^D^ 21.9 ± 0.9^D^ 17.2 ± 0.3^a^	15.6 ± 0.1^B^ 16.1 ± 0.1^BC^ 16.7 ± 0.2^ C^ 16.8 ± 0.2^ C^ 16.3 ± 0.1^b^	6.0 ± 0.1^E^ 7.4 ± 0.2^ F^ 10.7 ± 0.5^G^ 11.9 ± 0.6^G^ 8.2 ± 0.2^c^	12.8 ± 0.4^a^ 14.1 ± 0.4^a^ 15.8 ± 0.3^b^ 16.4 ± 0.3^b^ 14.8 ± 0.2

a–c: No difference between the main effects with the same letter; A–V: No difference between interactions that have the same letter; pruned average ± standard error

**Table 3d:** Descriptive statistics and multiple comparison results for ΔE_00_ values according to composite resin, group and time

Group	Time	Composite resin materials			Total
		FILTEK	CHARISMA	OMNICHROMA	
**Group 1**	ΔE_00 (1 day−baseline)_ ΔE_00 (7 days−baseline)_ ΔE_00 (30 days−baseline)_ ΔE_00 (Total)_	1.08 ± 0.18^ABCD^ 1.73 ± 0.14^ACEFG^ 1.12 ± 0.13^ABCD^ 1.35 ± 0.10^AB^	1.38 ± 0.14^ABCDEF^ 1.23 ± 0.15^ABCDE^ 1.55 ± 0.25^ABCDEF^ 1.37 ± 0.11^AB^	1.30 ± 0.09^ACDEF^ 1.17 ± 0.07^ABCDE^ 0.69 ± 0.12B^D^ 1.09 ± 0.06^B^	1.25 ± 0.06 1.36 ± 0.08 1.08 ± 0.09 1.24 ± 0.05^a^
**Group 2**	ΔE_00 (1 day−baseline)_ ΔE_00 (7 days−baseline)_ ΔE_00 (30 days−baseline)_ ΔE_00 (Total)_	2.95 ± 0.33^ACEFGHİ^ 7.64 ± 0.90^HİJKLMNOP^ 14.15 ± 1.57^JKLMO^ 7.36 ± 0.85^ C^	0.74 ± 0.06^B^ 1.70 ± 0.29^ABCDEFG^ 4.66 ± 0.52^GHİNP^ 2.06 ± 0.30^AB^	2.01 ± 0.14^CEFG^ 5.02 ± 0.30^HLNP^ 8.47 ± 0.28^JKM^ 5.08 ± 0.51^ C^	1.81 ± 0.17 4.74 ± 0.41 8.45 ± 0.61 4.53 ± 0.33^b^
**Group 3**	ΔE_00 (1 day−baseline)_ ΔE_00 (7 days−baseline)_ ΔE_00 (30 days−baseline)_ ΔE_00 (Total)_	2.01 ± 0.23^ABCDEFG^ 6.66 ± 0.48^JLNOP^ 12.89 ± 1.18^JKMO^ 6.68 ± 0.83^ C^	0.80 ± 0.09^BD^ 1.94 ± 0.16^ACEFG^ 3.34 ± 0.37^EFGHİ^ 1.95 ± 0.18^ A^	1.33 ± 0.07^ACDEF^ 4.81 ± 0.27^HLNP^ 8.83 ± 0.29^JKM^ 4.89 ± 0.59^ C^	1.36 ± 0.11 4.55 ± 0.36 8.45 ± 0.67 4.05 ± 0.34^b^
**Group 4**	ΔE_00 (1 day−baseline)_ ΔE_00 (7 days−baseline)_ ΔE_00 (30 days−baseline)_ ΔE_00 (Total)_	3.00 ± 0.36^ACEFGHİ^ 7.40 ± 0.75^HJKLNOP^ 14.03 ± 1.05^ M^ 7.70 ± 0.85^ C^	1.06 ± 0.09^ABD^ 2.33 ± 0.33^ABCDEFGİ^ 3.58 ± 0.39^FGHİN^ 2.16 ± 0.26^ A^	1.79 ± 0.11^CEFG^ 6.22 ± 0.27^LOP^ 10.22 ± 0.32^KM^ 5.88 ± 0.66^ C^	1.80 ± 0.12 5.48 ± 0.40 9.16 ± 0.76 4.73 ± 0.39^b^
**Total**	ΔE_00 (1 day−baseline)_ ΔE_00 (7 days−baseline)_ ΔE_00 (30 days−sbaseline)_ ΔE_00 (Total)_	2.23 ± 0.15^ A^ 5.97 ± 0.50^BC^ 10.98 ± 1.08^D^ 5.18 ± 0.41^a^	0.97 ± 0.05^E^ 1.80 ± 0.12^AF^ 3.27 ± 0.27^G^ 1.81 ± 0.11^b^	1.57 ± 0.07^ F^ 4.63 ± 0.33^BG^ 7.91 ± 0.65^CD^ 3.76 ± 0.29^c^	1.52 ± 0.06^a^ 3.78 ± 0.22^b^ 6.47 ± 0.41^c^ 3.21 ± 0.16

a–c: No difference between the main effects with the same letter; A–P: No difference between interactions that have the same letter; pruned average ± standard error

**Table 4:** Comparison of L*, a*, b*, and ΔE_00_ values according to composite resin, group and time

Variables	L*		a*		b*		ΔE_00_	
	**Test statistics**	***p***	**Test statistics**	***p***	**Test statistics**	***p***	**Test statistics**	***p***
Composite resin	2,157	**< 0.001**	42,840	**< 0.001**	5,173	**< 0.001**	652	**< 0.001**
Group	922	**< 0.001**	1,215	**< 0.001**	800	**< 0.001**	1,149	**< 0.001**
Time	1,611	**0.001**	1,094	**0.001**	756	**0.001**	857	**0.001**
Composite resin*Group	201	**0.001**	257	**0.001**	374	**0.001**	511	**0.001**
Composite resin *Time	428	**0.001**	669	**0.001**	242	**0.001**	292	**0.001**
Group*Time	834	**0.001**	679	**0.001**	605	**0.001**	717	**0.001**
Composite resin*Group*Time	196	**0.001**	408	**0.001**	180	**0.001**	221	**0.001**

*Robust ANOVA test, *p* < 0.005

**Table 5:** Descriptive statistics and multiple comparison results for roughness values according to composite resin, group and time

Group	Time	Composite resin materials			Total
		FILTEK	CHARISMA	OMNICHROMA	
**Group 1**	Baseline 1 day 7 days 30 days Total	0.210 ± 0.000 0.217 ± 0.002 0.218 ± 0.002 0.220 ± 0.003 0.216 ± 0.001^ABC^	0.228 ± 0.002 0.209 ± 0.005 0.222 ± 0.005 0.238 ± 0.003 0.224 ± 0.002^ABD^	0.118 ± 0.006 0.133 ± 0.008 0.130 ± 0.010 0.127 ± 0.007 0.127 ± 0.004 F	0.195 ± 0.010 0.195 ± 0.007 0.197 ± 0.009 0.209 ± 0.009 0.198 ± 0.004^a^
**Group 2**	Baseline 1 day 7 days 30 days Total	0.225 ± 0.013 0.230 ± 0.000 0.234 ± 0.023 0.253 ± 0.015 0.234 ± 0.005^D^	0.213 ± 0.018 0.222 ± 0.012 0.240 ± 0.013 0.256 ± 0.017 0.232 ± 0.008^ABD^	0.178 ± 0.026 0.196 ± 0.033 0.179 ± 0.008 0.191 ± 0.011 0.186 ± 0.010^BCE^	0.209 ± 0.011 0.219 ± 0.007 0.216 ± 0.009 0.234 ± 0.010 0.219 ± 0.005^b^
**Group 3**	Baseline 1 day 7 days 30 days Total	0.210 ± 0.048 0.259 ± 0.007 0.269 ± 0.041 0.266 ± 0.032 0.250 ± 0.018^ABCDE^	0.226 ± 0.009 0.234 ± 0.005 0.260 ± 0.013 0.263 ± 0.019 0.244 ± 0.007^D^	0.150 ± 0.020 0.178 ± 0.024 0.192 ± 0.016 0.196 ± 0.005 0.184 ± 0.008^E^	0.193 ± 0.013 0.231 ± 0.011 0.232 ± 0.014 0.233 ± 0.011 0.224 ± 0.006^b^
**Group 4**	Baseline 1 day 7 days 30 days Total	0.218 ± 0.015 0.297 ± 0.021 0.230 ± 0.006 0.236 ± 0.006 0.241 ± 0.007^AD^	0.215 ± 0.023 0.239 ± 0.015 0.241 ± 0.003 0.262 ± 0.004 0.245 ± 0.006^D^	0.148 ± 0.020 0.197 ± 0.005 0.186 ± 0.017 0.201 ± 0.017 0.186 ± 0.009^CE^	0.195 ± 0.014 0.237 ± 0.010 0.225 ± 0.008 0.239 ± 0.007 0.225 ± 0.005^b^
**Total**	Baseline 1 day 7 days 30 days Total	0.212 ± 0.008 0.238 ± 0.005 0.227 ± 0.007 0.236 ± 0.006 0.230 ± 0.003^a^	0.221 ± 0.006 0.224 ± 0.004 0.240 ± 0.004 0.251 ± 0.004 0.235 ± 0.003^a^	0.142 ± 0.009 0.170 ± 0.009 0.170 ± 0.007 0.180 ± 0.007 0.167 ± 0.004^b^	0.195 ± 0.006^a^ 0.217 ± 0.004^b^ 0.215 ± 0.005^b^ 0.226 ± 0.004^b^ 0.214 ± 0.002

a–b: No difference between the main effects with the same letter; A–F: No difference between the main effects with the same letter; pruned average ± standard error

**Table 6:** Test statistics for comparison of roughness values according to composite resin, group and time

Variables	Test statistics	*p*
Composite resin Group Time Composite resin*Group Composite resin*Time Group*Time Composite resin*Group*Time	178,030 91,690 17,420 25,070 9,670 16,750 10,520	**< 0.001** **< 0.001** **0.002** **0.001** 0.168 0.083 0.937

*Robust ANOVA test, *p* < 0.005

### Scanning electron microscopy (SEM) analysis

**Fig. 1:**
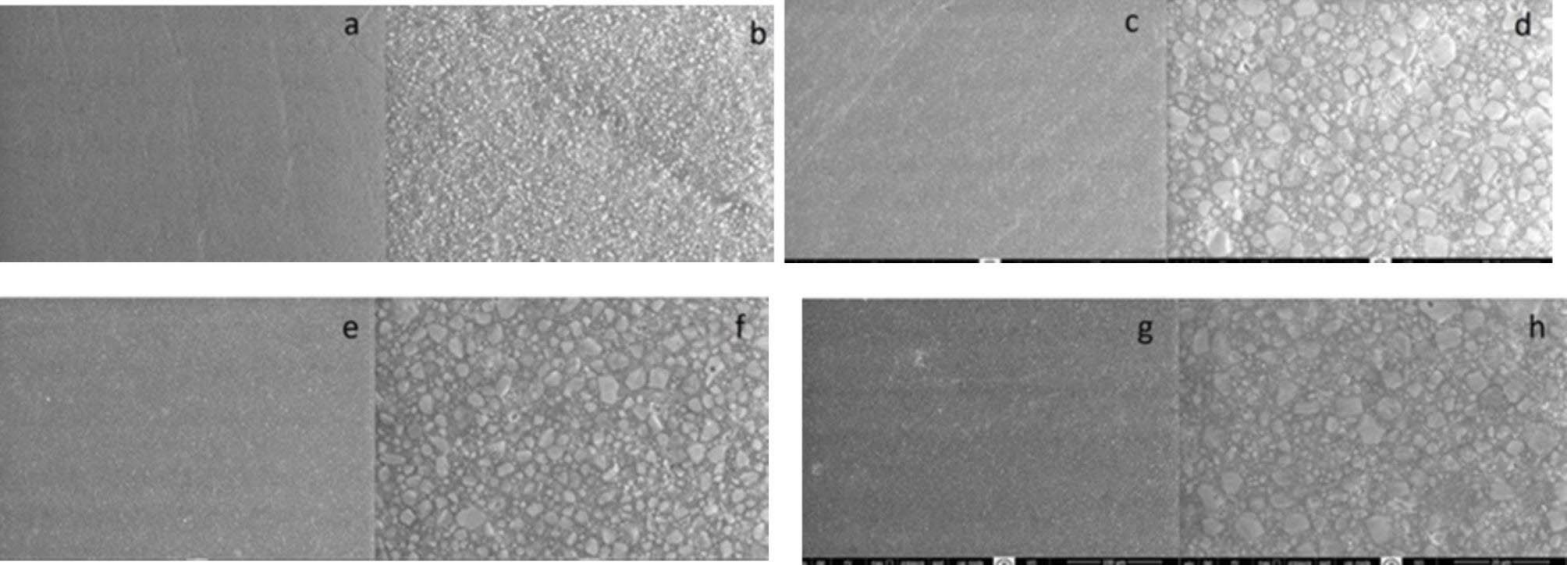
Representative images of Filtek Ultimate Universal composite resin surface by SEM after 30 days at ×1000 and ×5000 magnification: (**a–b**) Group 1 (control); (**c–d**) Group 2 (Colgate Total 12); (**e–f**) Group 3 (Colgate Optic White); and (**g–h**) Group 4 (Meridol Gentle White)

**Fig. 2:**
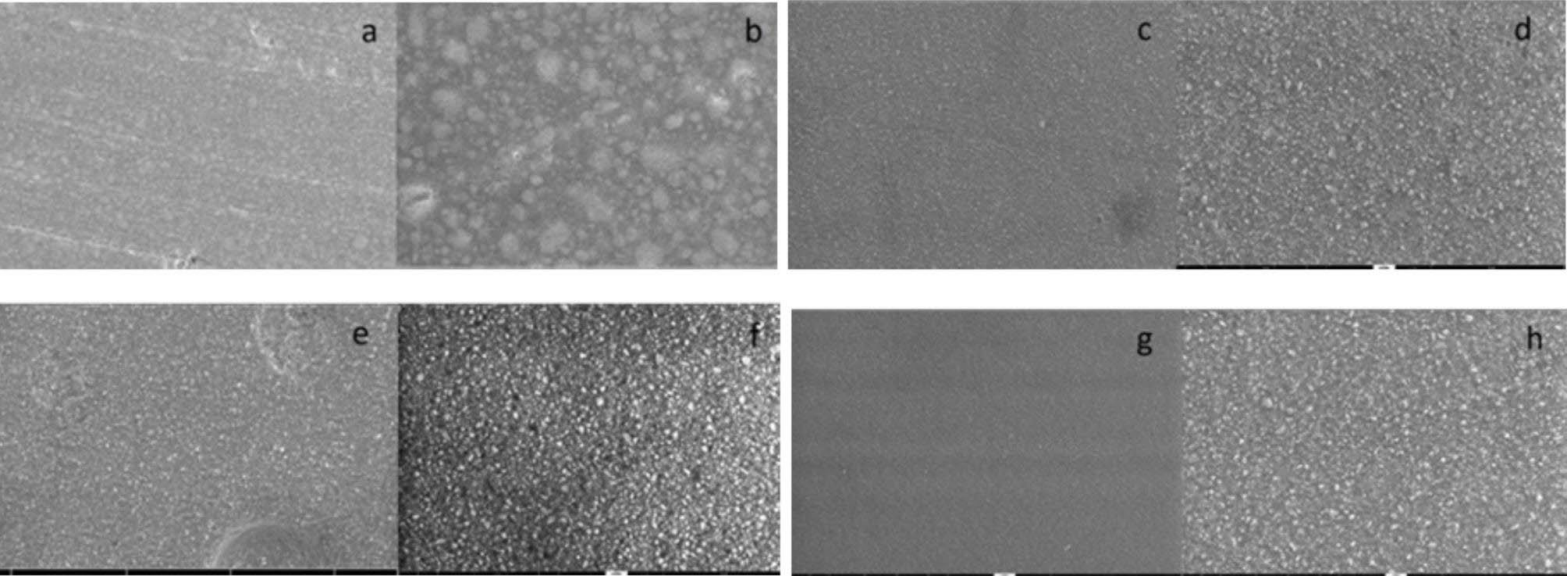
Representative images of Charisma Smart composite resin surface by SEM after 30 days at ×1000 and ×5000 magnification: (**a–b**) Group 1 (control); (**c–d**): Group 2 (Colgate Total 12); (**e–f**): Group 3 (Colgate Optic White); and (**g–h**) Group 4 (Meridol Gentle White)

**Fig. 3:**
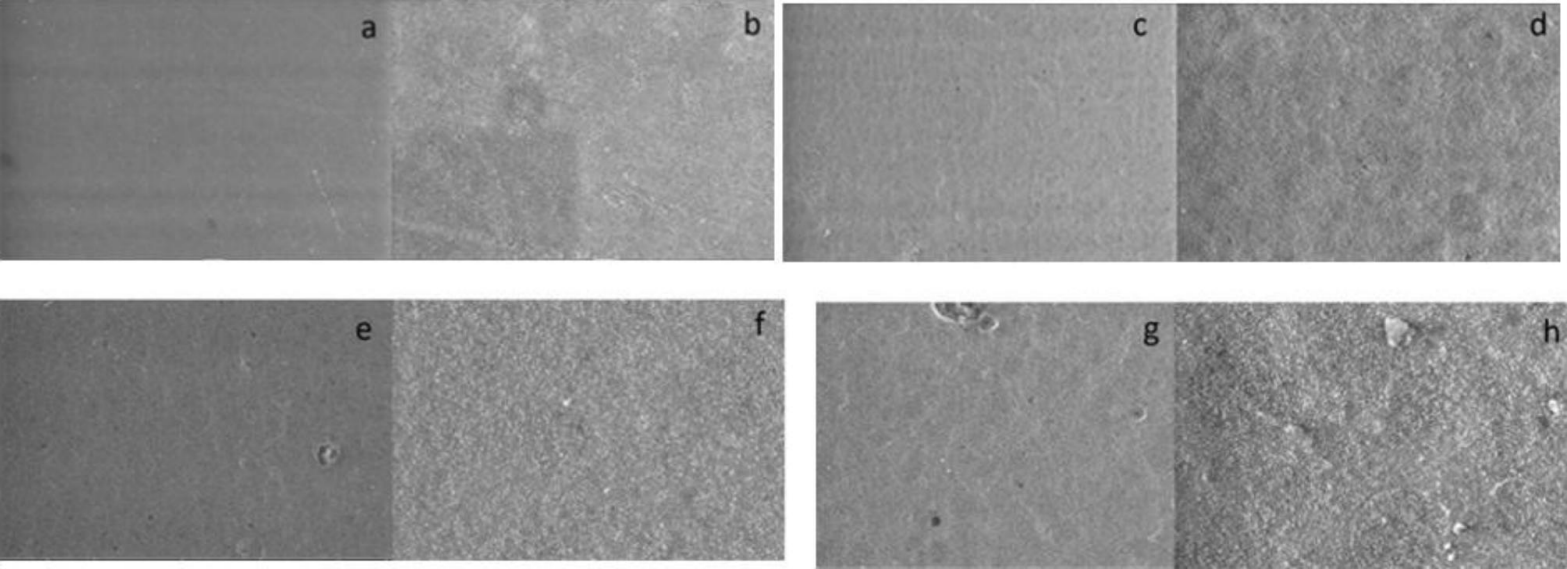
Representative images of Omnichroma composite resin surface by SEM after 30 days at ×1000 and ×5000 magnification: (**a–b**) Group 1 (control); (**c–d**) Group 2 (Colgate Total 12); (**e–f**) Group 3 (Colgate Optic White); and (**g–h**): Group 4 (Meridol Gentle White)

There were no cracks, fractures or ruptures of inorganic particles in the control and toothpaste groups of the tested composite resin materials (see Figs. 1, 2 and 3).

## Discussion

The results showed that the null hypothesis (i.e. that there would be no differences in color change and surface roughness of nano-hybrid, micro-hybrid and supra-nano-filled composite resin samples after brushing with whitening toothpastes) was unsupported.

This study examined the in vitro effects of brushing for 30 days with whitening toothpaste on the color and surface roughness of composite resins. The abrasiveness of the toothpaste can be measured using relative dentine abrasivity (RDA) [16]. Although the abrasives in toothpastes can prevent extrinsic staining of teeth, the abrasiveness of the toothpaste should be safe and at tolerable levels. It is recommended that the RDA of toothpaste not exceed 250 and that whitening toothpaste have an average RDA ranging between 60 and 100 or higher than 100, as detailed by the International Organization for Standardization (ISO) [16, 24, 25]. Therefore, for our study, we selected toothpastes with similar relative RDA rates and moderate abrasiveness.

We used a coffee solution for the staining because coffee (one of the most commonly used beverages) causes the greatest discoloration of restorative materials [19, 26, 27]. Moreover, the high temperature and acidity of coffee can cause composite resin discoloration. In addition to surface staining, coffee can cause subsurface staining due to its polar and delayed release colorants being absorbed by the composite resin surface [28]. Furthermore, scholars have reported that a greater temperature in the environment can hasten the discoloration of restorative materials. Therefore, composite resin samples were stored in an incubator at 37 ºC to stimulate the oral environment. The coffee solution was prepared by the investigator for every brushing cycle to minimise bacterial growth [19]. To deal with the problem of the visual assessment of color, devices such as colorimeters and spectrophotometers have been used in previous studies [19, 29]. According to the literature, color can be detected with a spectrophotometer device 33% more accurately and 93.3% more objectively than using the visual method [30]. Therefore, we performed color assessments with a digital spectrophotometer device.

The CIE L*a*b color system is generally used to measure color in dentistry [31]. The CIE L*a*b system can provide a standardised technique for measuring ΔE* values in an accurate manner. The small color changes identified by this system offer the advantages of improved objectivity, repeatability and sensitivity [29]. However, we selected the CIEDE 2000 color difference formula (ΔE_00_) to calculate single-number shade pass and avoid failures in evaluating minor to medium color disparities, rather than the previous CIE L*a*b system [19]. Scholars have found that color variation can be determined using perceptibility and acceptability thresholds. In our study, the perceptibility (ΔE_00_) and 50:50% acceptability thresholds were set at 0.8 and 1.8, respectively [19, 31]. Finally, in the present study, the perceptibility and acceptability values were higher after 30 days for all composite resin materials, which correlated with the previous study performed by Rohym et al. [19].

The organic components and filler particle properties of composite resin materials cause discoloration [32]. Aggregated filler and glass particles are vulnerable to porosities, with water absorption leading to staining and color changes in the nano-filled composite resin [33, 34]. Therefore, triethylene glycol dimethacrylate (TEGDMA) leads to further deterioration of the matrix/fill particle bond and brushing this composite resin may lead to further removal of these particles from the surface and thus to reduced color stability in the nano-filled composite resin material [1, 35]. In a study examining color change in nano-hybrid and micro-hybrid composite resins, the authors reported that the greatest color change occurred in the nano-hybrid composite resin containing TEGDMA [36]. In another study, the color stability and surface roughness of nano-filled, nano-hybrid, and micro-hybrid composite resins were reduced after brushing with whitening toothpaste, and it was reported that the greatest color change occurred in the nano-hybrid composite resin material; these results correlate with our findings. Furthermore, the same study reported that the late penetration of food-simulating substances through the polymer matrix may cause discoloration in the nano-hybrid composite resin [37]. Similar to the literature, in our study, the greatest discoloration was found in the nano-hybrid composite resin (Filtek Ultimate Universal, 3 M/ESPE, Saint Paul, USA). This may be due to the type and amount of filler particles in the composite resin material. We believe that the TEGDMA ratio of the nano-hybrid composite resin may lead to increased water absorption and increased polymer solubility [38].

It is known that there is a significant difference between the nano-hybrid and micro-hybrid composite resins in terms of filler size. According to the manufacturer, the nano-filled composite resin contains nanoclusters consisting of zirconia (4–11 nm) and silica (20 nm) nanoparticles, while the micro-hybrid composite resin contains micro-glass particles. Nanoclusters have been noted to have micropores that facilitate fluid absorption and pigment retention. In addition, nanoparticles contain large amounts of atomic particles on their surfaces [36]. It has been said that the quantum effect changes with the size of the particle and that the shrinking of particle size leads to the effects becoming apparent. The quantum effect of nanoparticles exposes them to their simple agglomeration within dust particles, thereby making them susceptible to different surface interactions, including the adsorption of other substances [39]. In line with the literature, our study revealed that the color change of the nano-hybrid composite resin occurred at a higher rate than that of the micro-hybrid composite resin.

Furthermore, scholars have reported that the nano-hybrid composite resin is more unstable in terms of color than the supra-nano-filled composite resin (Omnichroma), which correlated with our results [40]. The lower filler content and presence of nanoclusters can explain the lower color resistance of the nano-hybrid composite resin compared to the supra-nano-filled resin composite (Omnichroma) [19, 41, 42]. In this study, we evaluated the effects of toothpaste on the color stability of composite resins rather than evaluating the coloration of composite resins. We found that traditional toothpaste, peroxide-based toothpaste and blue covarine-based toothpaste could not prevent the coloration of the coffee-colored supra-nano-filled composite resin samples (Omnichroma).

The literature states that filler size and surface roughness are not affected by tooth brushing, but the average surface roughness value generally increases in composite resins with larger filler sizes [9]. This is explained by the gradual removal of fillers after tooth brushing. The larger the filler size, the more filler will be removed and the more the surface roughness of the material will increase. However, the shape of the filler, the distance between the fillers, the composite resin matrix composition, the chemical bond between the filler particles and the degree of conversion after polymerisation are factors that must be considered [43]. Scholars have reported that there is a material-dependent interaction between toothpaste abrasiveness and the surface roughness of restorative materials [44]. Various studies have determined that the surface roughness values for micro-hybrid composite resins after the use of whitening paste are higher than for nanocomposite resin systems, as supported by our findings [43–45]. In our study, although there was no statistically significant difference between the surface roughness values of the micro-hybrid composite resin and the nano-hybrid composite resin after 30 days of brushing with whitening toothpaste, the numerical values for the micro-hybrid composite resin were higher than those of the nanohybrid composite resin. In addition, in the current study, the lowest surface roughness values belonged to the supra-nano-filled composite resin.

In addition, it has been mentioned in the literature that surface roughness can cause the external discoloration of composite resin materials [19, 41]. The wearing out of the resin composite can lead to the debonding of the inorganic fillers from the resin matrix, which can cause voids and increase surface roughness, thereby creating a surface that is susceptible to external stains [41, 46]. Moreover, the resin matrix is a key element in staining susceptibility [22, 29, 47]. The discoloration of the resin matrix depends on the hydrophilicity of the resin matrix and the water absorption of the material, as indicated in several studies [19, 46, 48]. Moreover, our finding that the Omnichroma composite resin containing a matrix composition based on TEGDMA and urethane dimethacrylate (UDMA) was more discolored than the Charisma composite resin correlated with the results of previous studies [19, 42, 49]. Although we evaluated the effects of whitening toothpaste on nano-hybrid, supra-nano-filled and micro-filled composite resin materials, our study had limitations. More specifically, we could not mimic the factors affecting the restorative materials in the oral cavity, such as microbiota, salivary circulation, temperature and pH changes, as done in other in vitro studies [19, 49]. Therefore, it was not possible to precisely simulate the conditions of the oral cavity.

## Conclusion

Within the limitations of the present study, the findings showed after 30 sequential days, traditional and whitening toothpastes could not decrease discoloration on the micro-hybrid, nano-filled and supra-nano-filled composite resin samples caused by the coffee solution to the level below the perceptibility threshold. The smallest color change was observed in the micro-hybrid composite resin and the greatest color change was observed in the nano-hybrid composite resin. The greatest color change was obtained after using a toothpaste based on blue covarine, while the smallest color change was observed after using peroxide-based toothpaste. The supra-nano-filled composite resin samples had the lowest surface roughness values compared to the micro-hybrid and nano-filled composite resin samples. Additional laboratory and clinical studies are needed to fully understand the long-term effectiveness of whitening toothpaste on composite resin materials.

## Data Availability

The datasets used and/or analysed in this study are available from the corresponding author upon reasonable request.
